# Computer Simulations of the Temperature Dependence of Enzyme Reactions

**DOI:** 10.1021/acs.jctc.4c01733

**Published:** 2025-01-30

**Authors:** Johan Åqvist, Bjørn O. Brandsdal

**Affiliations:** † Department of Cell & Molecular Biology, Uppsala University, Biomedical Center, SE-751 24 Uppsala, Sweden; ‡ Department of Chemistry, University of Tromsø − The Arctic University of Norway, N9037 Tromsø, Norway

## Abstract

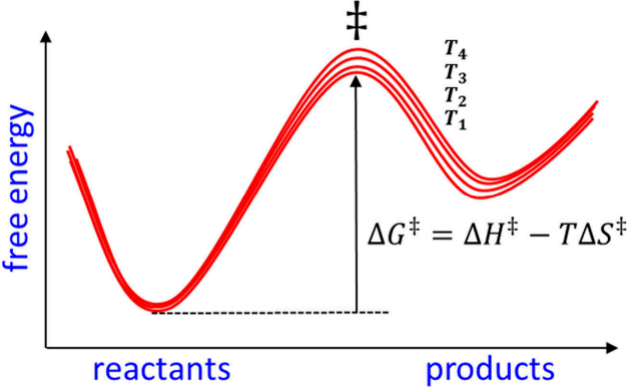

In this review we discuss the development of methodology for calculating the temperature dependence and thermodynamic activation parameters for chemical reactions in solution and in enzymes, from computer simulations. We outline how this is done by combining the empirical valence bond method with molecular dynamics free energy simulations. In favorable cases it turns out that such simulations can even capture temperature optima for the catalytic rate. The approach turns out be very useful both for addressing questions regarding the roles of enthalpic and entropic effects in specific enzymes and also for attacking evolutionary problems regarding enzyme adaptation to different temperature regimes. In the latter case, we focus on cold-adaptation of enzymes from psychrophilic species and show how computer simulations have revealed the basic mechanisms behind such adaptation. Understanding these mechanisms also opens up the possibility of designing the temperature dependence, and we highlight a recent example of this.

## Introduction

The temperature dependence of enzyme catalysis is a very old topic that in recent years has received renewed attention. One of the reasons for this is that observed Arrhenius behavior of catalytic rates allows for disentangling enthalpic and entropic effects in enzyme catalysis, which have long been the subject of debates.^1−8^ Another reason is that enzymes which display non-Arrhenius behavior (nonlinear Arrhenius plots) are equally interesting since the origin of such effects still remains elusive in many cases. Moreover, it has nowadays become possible to explore the temperature dependence of enzymatic rates by computer simulations that reliably evaluate activation free energies as a function of temperature. This allows for unprecedented insight into how enthalpic and entropic factors are manifested on a microscopic level and what their structural origins are. In this review, we will briefly outline the procedures for calculating the temperature dependence of chemical free energy barriers in enzymes and highlight how this can be used to address several important questions related to enzyme mechanism and evolution.

Since it is rarely possible to use brute force molecular dynamics (MD) simulations to directly observe reactive events in enzymes, i.e., to actually count the number of barrier crossings, the focus has to be on calculations of free energy barriers. This can be done with free energy perturbation (FEP) and umbrella sampling techniques in combination with MD, provided that the underlying reactive potential energy surface is known. For a system obeying Arrhenius behavior, the idea is then to obtain the temperature dependence of the first-order rate constant from the transition state theory equation
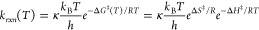
1where κ is the transmission coefficient (most often assumed to be unity for condensed phase reactions), *k*_*B*_ and *h* are Boltzmann’s and Planck’s constants, respectively, and Δ*G*^‡^(*T*) is the activation free energy at a given temperature. By calculating Δ*G*^‡^(*T*) at a series of different temperatures, its enthalpic (Δ*H*^‡^) and entropic () components can be obtained from linear regression using either Δ*G*^‡^ (T) =  or  =  This is what is usually called Arrhenius or Eyring plots and which of the expressions that is best for fitting the data depends on the magnitudes of Δ*H*^‡^ and Δ*S*^‡^. That is, one chooses the plot with the largest slope in order for *R*^2^ to be most meaningful.

How then do we represent the potential energy surface of the chemical reaction, that should properly take into account bond breaking and formation and change of the charge distribution of the reacting groups? Here, it is important to keep in mind that the thermodynamic activation parameters are “global” properties of the reacting system and thus include contributions from both the enzyme and substrate, as well as the surrounding water solvent. Hence, a sufficiently large system surrounding the enzyme active site is clearly needed and that prompts for a QM/MM representation of the system. An additional complication is that the evaluation of Δ*G*^‡^ and even more so of Δ*H*^‡^ and Δ*S*^‡^, requires a substantial amount of configurational sampling in order to achieve convergence of the calculated properties. This makes the empirical valence bond (EVB) method^2,9^ the most suitable for this kind of studies, since it allows for massive sampling by MD simulation due to its simple force field character. With MD/EVB simulations it is possible to carry out hundreds of replicate simulations and to do this at different well-defined temperatures, to achieve sampling on the microsecond scale. However, the approach requires detailed data on the reaction mechanism for its parametrization and it is therefore often complemented with standard quantum mechanical calculations. With regard to calculations of enzyme Arrhenius plots, there have also recently been a few reports on QM/MM approaches based either on semiempirical AM1 or DFTB2 models,^10,11^ but higher level Hamiltonians and density functionals seem too expensive at present. We will briefly outline the EVB procedure below.

## Calculating the *T*-Dependence with EVB

**Figure 1:**
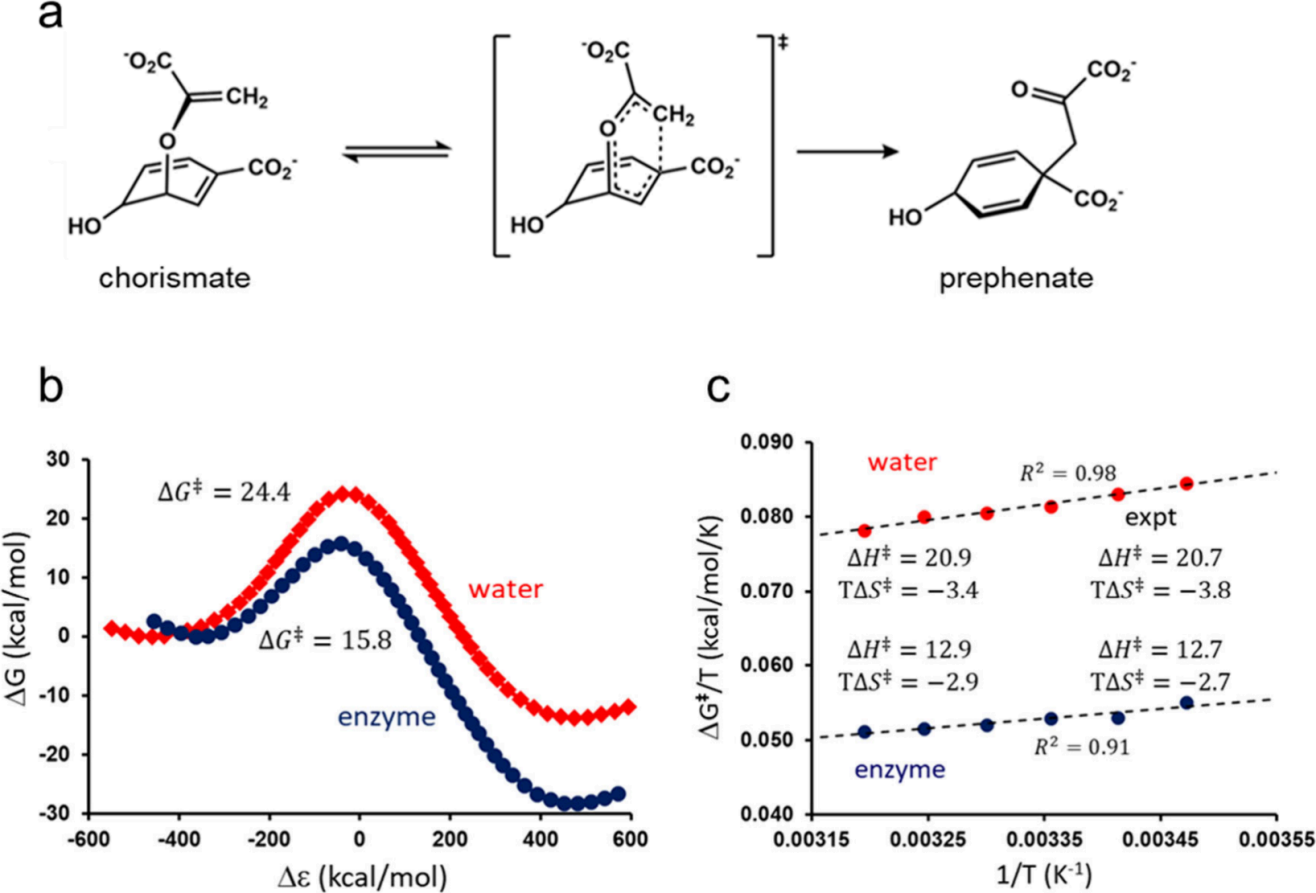
(**a**) The rearrangement of chorismate to prephenate catalyzed by the chorismate mutase. (**b**) Calculated reaction free energy profiles for uncatalyzed reaction in water (red) and for the *B. subtilis* chorismate mutase catalyzed reaction (blue) at 25 °C. The generalized reaction coordinate Δε is the energy gap between the two diabatic potentials for reactant and product.^2,9^ (**c**) Computed Arrhenius plots for the two reactions from MD/EVB simulations at six temperatures.^12^

As an example here, we consider the pericyclic rearrangement of chorismate to prephenate catalyzed by chorismate mutase, as well as the corresponding spontaneous uncatalyzed reaction in water (Figure 1a). This is a particularly simple reaction since it is unimolecular and no consideration of concentration effects associated with bringing reactants together are required. The reaction can be described by a two-state EVB model and the corresponding Hamiltonian matrix
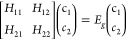
2can be parametrized directly from density functional theory (DFT) calculations on the uncatalyzed reaction with a continuum solvent model.^12^ The diagonal matrix (*H*_*ii*_) elements are the reactant and product potential energies and are represented by a standard force field (OPLS-AA/M and TIP3P water).^13,14^ Their absolute energy difference, with the reacting groups at equilibrium geometries and noninteracting, is fitted to reproduce the DFT exothermicity by adding an energy constant (Δα) to *H*_22_. This is necessary since regular force fields do not know about heats of formation. The off-diagonal terms (*H*_12_ = *H*_21_) are also fitted to the DFT results to reproduce the calculated activation barrier.^15^

In this example, it turns out that the B3LYP functional^16^ (B3LYP/6-311G+(2d,2p)) with the CPCM solvation model^17^ gives perfect agreement with experimental results for the reaction in water at 298 K ( kcal/mol)^18^ and the predicted exothermicity is −13.2 kcal/mol.^12^ Hence, by running MD simulations with the free energy perturbation method to drive the reaction from reactant to product, the ground-state free energy profile of the reaction in water is obtained as described in ref (15). The goal is thus to make the free energy (Δ*G*_*g*_) on the ground-state potential (*E*_*g*_) reproduce the DFT values of Δ*G*^‡^ and Δ*G*^0^ (Figure 1b). This can conveniently be done after the MD trajectories have been generated with FEP simulations, simply by varying Δα and *H*_12_ in the subsequent free energy calculation. In practice, 300 replicate MD/FEP simulations with different initial conditions were run at 298 K with the reacting molecule immersed in a 50 Å diameter sphere of water, to yield 153 ns of sampling. For evaluation of the temperature dependence, it is essential to have sufficient sampling so that the errors in the calculated Δ*G*^‡^ can be brought down to about 0.1–0.2 kcal/mol.^19^

To obtain estimates of Δ*H*^‡^ and Δ*S*^‡^ one then just repeats the above simulations at a series of different temperatures and constructs Arrhenius plots from the data, from which the thermodynamic activation parameters are obtained by linear regression. One can then either plot Δ*G*^‡^ vs *T* or Δ*G*^‡^/*T* vs 1/*T*, as discussed above. It is, of course, essential that the temperature is well-defined and any thermostat that gives correct mean kinetic energy can be used.^20^ Most often the Berendsen thermostat^21^ has been used with a relatively strong bath coupling (τ = 10–100 fs) and separate scaling for protein and water. The calculated Arrhenius plot for the conversion of chorismate to prephenate in water obtained in this way is shown in Figure 1c, and the values of Δ*H*^‡^ and Δ*S*^‡^ are found to be excellent agreement with experimental data.^18^ Hence, calculated values are Δ*H*^‡^ = 20.9 and *T*Δ*S*^‡^ = −3.4 kcal/mol and experimental ones are Δ*H*^‡^ = 20.7 and TΔ*S*^‡^ = −3.8 kcal/mol (25 °C). The same approach has been used to study different possible pathways for the spontaneous deamination of cytidine in water, where the magnitudes of Δ*H*^‡^ and Δ*S*^‡^ could actually be used to determine the operational mechanism.^19^

The next step is then to put the reactant into the active site of the *Bacillus subtilis* enzyme chorismate mutase and run the analogous simulations of the enzyme system. In this case, a larger system with 72 Å diameter was used to have the entire enzyme solvated and the simulations were repeated 100 times at each temperature in the range 288–313 K.^12^ The results from these simulations are also shown in Figure 1b,c and, as expected, it is evident that the enzyme significantly reduces both Δ*G*^‡^ and Δ*G*^0^ compared to the reaction in solution (Δ*G*^0^ is the on-enzyme equilibrium free energy). The value of Δ*G*^‡^ = 15.8 kcal/mol obtained from the simulations at 298 K is also in very good agreement with the experimentally derived value of  kcal/mol. Moreover, the Δ*H*^‡^ and Δ*S*^‡^ estimates resulting from the computed Arrhenius plots are in strikingly good agreement with experiment, also for the enzyme reaction (Figure 1c). Hence, this example shows that the general MD//EVB approach for computing thermodynamic activation parameters is indeed reliable. Here, it should be noted that no information whatsoever regarding Δ*H*^‡^ and Δ*S*^‡^ goes into the parametrization of the uncatalyzed water reaction. That is, only the free energy barrier Δ*G*^‡^ is used in calibration of the EVB potential. As has been explained elsewhere,^15^ the fact that these parameters are well reproduced stem from the accuracy of the underlying force field (OPLS-AA/M).^13^ What is also clear from these simulations is that chorismate mutase primarily works by reducing the activation enthalpy of the reaction, which has been seen to originate from a more compact transition state (TS) enforced by the efficient enzyme preorganization.^12^ Chorismate mutase is thus a case where entropy plays a relatively minor role for the catalytic effect, but as we shall see below there are indeed cases where entropic effects dominate catalysis.

## EVB Parametrization for More Complex Reactions

Historically the EVB model for enzyme reactions was often parametrized on experimental data for Δ*G*^‡^ and Δ*G*^0^ for uncatalyzed solution reactions.^2,9^ This involves two problems, namely, (1) that the spontaneous reaction in water may follow a different mechanism from that in the enzyme and (2) that for bimolecular reactions, the free energy of bringing reactants together in water must be taken into account (sometimes called the “cage effect”).^2,4^ The second problem is rather trivial and is usually solved by considering the free energy change of bringing the reactants into contact (from a 1 M standard state) to be dominated by entropy. This leads to the common approximation that the corresponding free energy cost is , which reflects a reduction of the volume of one mole from 1 L to 1/55 L, where 55 M is the concentration of the surrounding water molecules.

We recently examined the reliability of the above approximation for the solution reaction corresponding to that catalyzed by the enzyme ketosteroid isomerase.^15^ The process considered in water is then the acetate catalyzed deprotonation of the steroid 5-androstene-3,17-dione, for which reliable experimental data for thermodynamic activation and equilibrium parameters are available.^22^ It turns out that fitting a two-state EVB model to the experimental values of Δ*G*^‡^ and Δ*G*^0^, and applying the  correction to the former (it cancels for Δ*G*^0^), delivers Δ*H* and Δ*S* values in excellent agreement with experiment, both for forward and reverse activation barriers and for the equilibrium constant.^15^ Hence, this approach can be considered viable, provided that the enzyme reaction to be studied follows that of an existing reference reaction in water.

**Figure 2:**
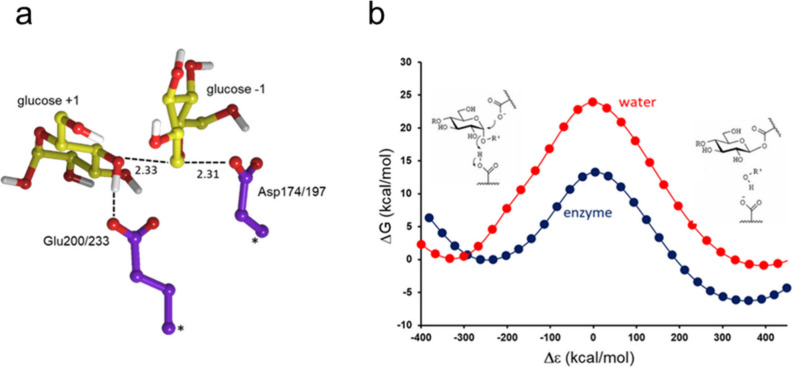
(**a**) Optimized transition-state structure for the reference reaction in water from DFT calculations (atoms marked with asterisks were fixed). (**b**) Calculated reaction free energy profiles for the reference reaction in water (red) and the reaction catalyzed by the cold-adapted α-amylase AHA (blue).^23^

But what to do when there is no spontaneous water reaction that follows the same pathway as in the enzyme and, thus, no experimental data is available? Here, there are basically three alternatives that have been used. The first is to use quantum mechanical calculations with a solvent model, just as for the chorismate reaction above, and evaluate the same mechanism in water that is followed in the enzyme. An example of this is the glycosidic bond cleavage in α-amylases, that hydrolyze starch molecules.^23^ In this case, the energetics of a small DFT model with continuum solvation, encompassing a glucose disaccharide and two carboxylic side chains corresponding to the catalytic groups, was evaluated as a model for the water reference reaction (Figure 2). When parametrizing an EVB model against this data and transferring it into the enzyme active site the thermodynamic activation parameters (Δ*G*^‡^, Δ*H*^‡^, Δ*S*^‡^) obtained from computational Arrhenius plots all turned out to agree well with experimental data.^24^ The same approach was also used to study the reactions of purine nucleoside phosphorylase with different substrates.^25^ It should be noted that when parametrizing EVB against solution data (experimental or from DFT) the overall catalytic effect of the enzyme compared to water can be directly assessed and its origin further examined. This is, of course, of major interest if one wants to understand where the catalytic effect of the enzyme comes from.

However, in some cases the enzyme mechanism is so complex that a simple reference reaction in water, with just a few active groups, becomes relatively meaningless. In such cases one has to resort to quantum mechanical calculations on a larger model of the active site and parametrize EVB directly on this model for the enzyme, while not worrying about any reference reaction. The two main options for this approach is to either use a large cluster model^26^ of the enzyme active site including water molecules, or to use QM/MM calculations that allow even larger models to be used. We have used both of these strategies for parametrizing EVB in the case of cytidine deaminase,^7^ hydroxybutyrate dehydrogenase (HBDH)^27,28^ and bacterial lipases (to be published). In these cases, subsequent MD/EVB simulations have delivered computational Arrhenius plots of good accuracy compared to experimental data, but this is of course dependent on that the cluster or QM/MM model yields reasonable activation barriers. For example, in the case of HBDH exponential averaging of 10 QM/MM minimum energy paths were used to obtain estimated values of  and  3.9 kcal/mol, which compares well with the experimental free energy barrier of  kcal/mol.^27^ The EVB model for HBDH was then directly parametrized on these enzyme values and MD/EVB simulations then delivered activation parameters of  and  kcal/mol, compared to experimental values of  and  kcal/mol (10 °C).^28^

## Why Do We Calculate Thermodynamic Activation Parameters?

There are several reasons for why it is of considerable interest to obtain, not only activation free energies, but also enthalpies and entropies from enzyme computer simulations. To understand the energetic origin of catalytic effects it has long been realized that the partitioning of activation free energies into their enthalpic and entropic components contains valuable information about what is going on at the microscopic level. For example, there have been many debates regarding the role of entropic versus enthalpic effects in enzyme catalysis and these issues are often difficult to settle by experimental means. That is, it is often difficult to establish the connection between enzyme structure and thermodynamics by experiment, while computer simulations may provide a more straightforward route to structure–function relationships. A typical example is the chorismate mutase reaction above, where EVB simulations could both confirm the strong enthalpic effect and also elucidate its origins.

**Figure 3:**
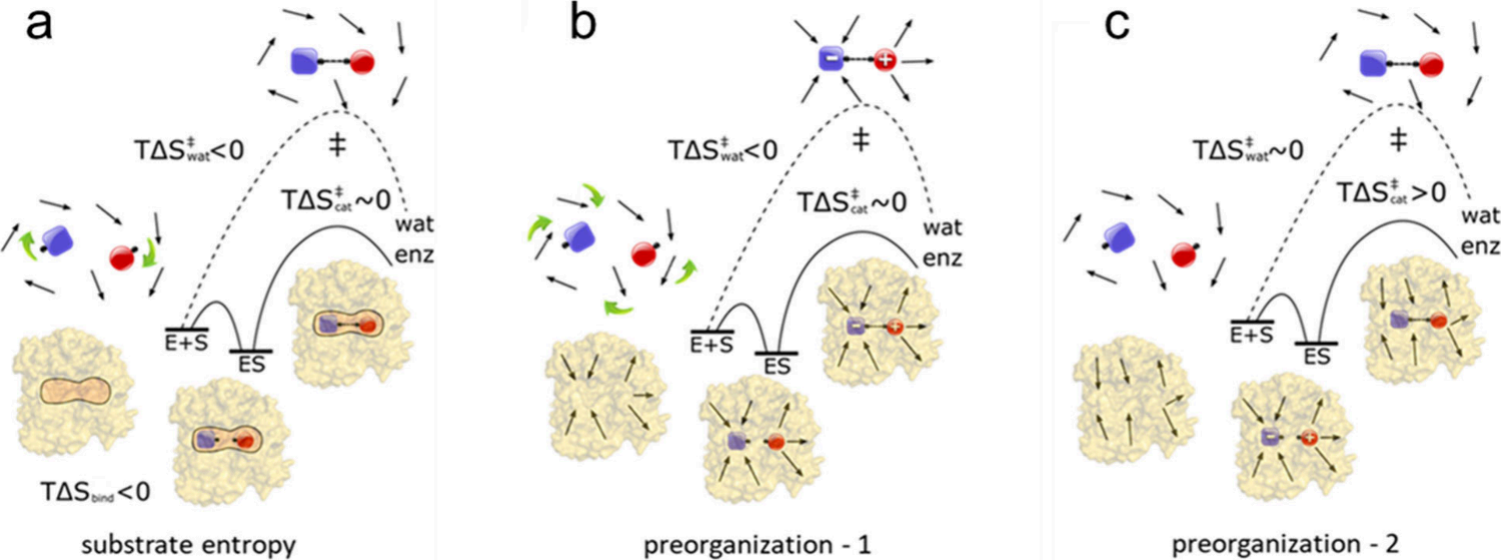
Three different ways in which an enzyme (enz) could reduce the entropy penalty compared to the uncatalyzed water reaction (wat).^36^ (**a**) If the entropy penalty of the uncatalyzed water reaction () is dominated by reorientation of the substrates, then the enzyme could pay the penalty already when binding them in a reactive orientation ( and ). (**b**) If  is dominated by reorientation of water molecules, then the enzyme could bind the substrates in a preorganized active site that does not need reorientation of the protein dipoles (). (**c**) If the uncatalyzed reaction has similarly small charge separation in the reactant and transition states, then the enzyme could yield a favorable activation entropy by stabilizing a more polar reactant state, causing a relaxation of the aligned protein dipoles in the transition state ().

A longstanding problem has also been to explain the origin of large favorable entropic effects in enzyme catalysis. Although statistics show that catalysis in most enzymes seems to be achieved by a reduction of Δ*H*^‡^,^5^ there are many examples of cases where Δ*S*^‡^ is significantly less negative than in typical uncatalyzed reference reactions and thus a major contributor to the catalytic effect.^29−31^ Early analyses of this problem were often focused on the conformational entropies of the substrates themselves. The prevailing idea was then that translational, rotational and conformational degrees of freedom of the substrates were “frozen out” upon binding to the enzyme active site.^1^ If the entropic barrier of the uncatalyzed reaction in water is dominated by a reduction of substrate entropy (bringing the reactants together in a productive alignment), then the enzyme could pay this penalty already when the substrates bind and position them for reaction in the ES complex (Figure 3a). The signature of such an entropy effect would then be a negative entropy of binding that is of similar magnitude to entropy penalty reduction for the chemical step  ∼  − 

Another explanation for a reduced −*TΔS*^‡^ penalty is the concept of active site preorganization, as outlined by Warshel.^32,33^ That is, if the entropy penalty for the uncatalyzed reaction is rather associated with reorganization of surrounding water molecules in the TS, than with the reactants themselves, then this penalty could be reduced by preorganizing the dipoles in the enzyme active site toward the TS configuration (Figure 3b). The idea that enzymes preorganize their active site also implies that they have to pay a price in terms of their folding energy or stability.^32,33^ This was, in fact, elegantly demonstrated experimentally by Matthews and co-workers in 1995, who showed that a series of mutations in the active site of T4-lysozyme increased stability of the protein while reducing activity.^34^ The preorganization concept as a cause of a reduced entropy penalty is now widely accepted and a pertinent example is the peptide bond formation reaction on the ribosome. There, the huge  kcal/mol penalty of the uncatalyzed water reaction is entirely abolished when the reaction takes place in the peptidyl transfer site of the ribosome.^30,31^ We could explain this effect in terms of a preorganized hydrogen bonding network involving ribosomal groups and water molecules and estimated a very large entropy penalty reduction for the reaction.^35^ An important experimental signature of preorganization in this case is also that both  and *k*_cat_ have similar near-zero entropies of activation, implying that substrate binding (A-site tRNA in this case) does not involve a large negative binding entropy.

An enzyme that appeared to obey the first type of entropy effect above, elimination of the entropy penalty upon substrate binding,^29^ is cytidine deaminase. Here,  kcal/mol closely matches the enthalpy penalty reduction for *k*_cat_ of  kcal/mol at room temperature.^29^ The huge catalytic effect of the enzyme (10^12^) is, in fact, dominated by the entropic effect, while the reduction of  is somewhat smaller (7 kcal/mol).^29^ In this case, the hydrolytic deamination reaction of cytidine is a rather complex process involving six distinct steps in the enzyme, where a catalytic zinc ion plays a major role. We therefore first used a DTF cluster model of the enzyme involving about 200 atoms to follow the catalytic process (B3LYP/6-311+G(2d,2p), LANL2TZ pseudopotential for Zn and CPCM solvation).^7^ An EVB model for all these steps was then constructed by fitting to the DFT results, which allowed Arrhenius plots to be calculated for each step from MD/EVB simulations. The calculations revealed that the rate-limiting step(s) in the enzyme has an activation entropy close to zero and a value of  kcal/mol, in agreement with the experimental results.^7^ However, the large reduction of the activation entropy penalty compared to the uncatalyzed water reaction was found to be due to a change of mechanism, which has nothing to do with substrate binding. That is, the enzyme-bound Zn^2+^ ion enforces a hydroxide attack mechanism, while the solution reaction was earlier found to involve a neutral water attack on the substrate. The OH^–^ attack mechanism was found to be associated with a favorable  also in water, although it is not preferred in solution.^19^ The underlying reason for this effect is that the charge on OH^–^ is more concentrated (localized) than in the transition state for nucleophilic attack, where it becomes more delocalized. This leads to a relaxation of the dipolar environment as the TS is approached with a concomitant entropy increase (Figure 3c).^7^ A similar effect, where the enzyme environment stabilizes a more polar reactant state than in solution, leading to a positive , was also found in simulations of GTP hydrolysis by EF-Tu on the ribosome.^36^

The above examples are intended to show how computer simulations of the temperature dependence of enzyme reactions can be used to address some classical questions in enzymology regarding the roles of enthalpy and entropy. However, as it turns out, the same computational approach can also be used to address evolutionary questions about how enzymes have been adapted to different thermal environments. The key idea here is then to carry out comparative simulations of orthologous enzymes from differently adapted species.

## Enzyme Cold-Adaptation

A most remarkable biochemical finding was reported by George Somero and co-workers more than 50 years ago.^37^ By measuring the kinetics of three different enzymes purified from warm- and cold-blooded species (rabbit, chicken, tuna, halibut, lobster, cod) at different temperatures, a consistent trend was found regarding activation enthalpies and entropies. All the cold-active marine enzymes had a markedly lower value of  and a more negative  than the enzymes from warm-blooded species and were also consistently faster, particularly at low temperature. This finding marked the start of biochemical studies of enzyme cold-adaptation and it subsequently turned out that the shift in enthalpy–entropy balance was a universal phenomenon.^38,39^ So, the question naturally arose: what is the structural origin of this phenomenon? It should be noted here that comparative studies of enzymes from phsychrophilic (cold-adapted), mesophilic and thermophilic species pertain to orthologous enzymes that have very similar 3D structures and, ideally, also high sequence identity.

**Figure 4:**
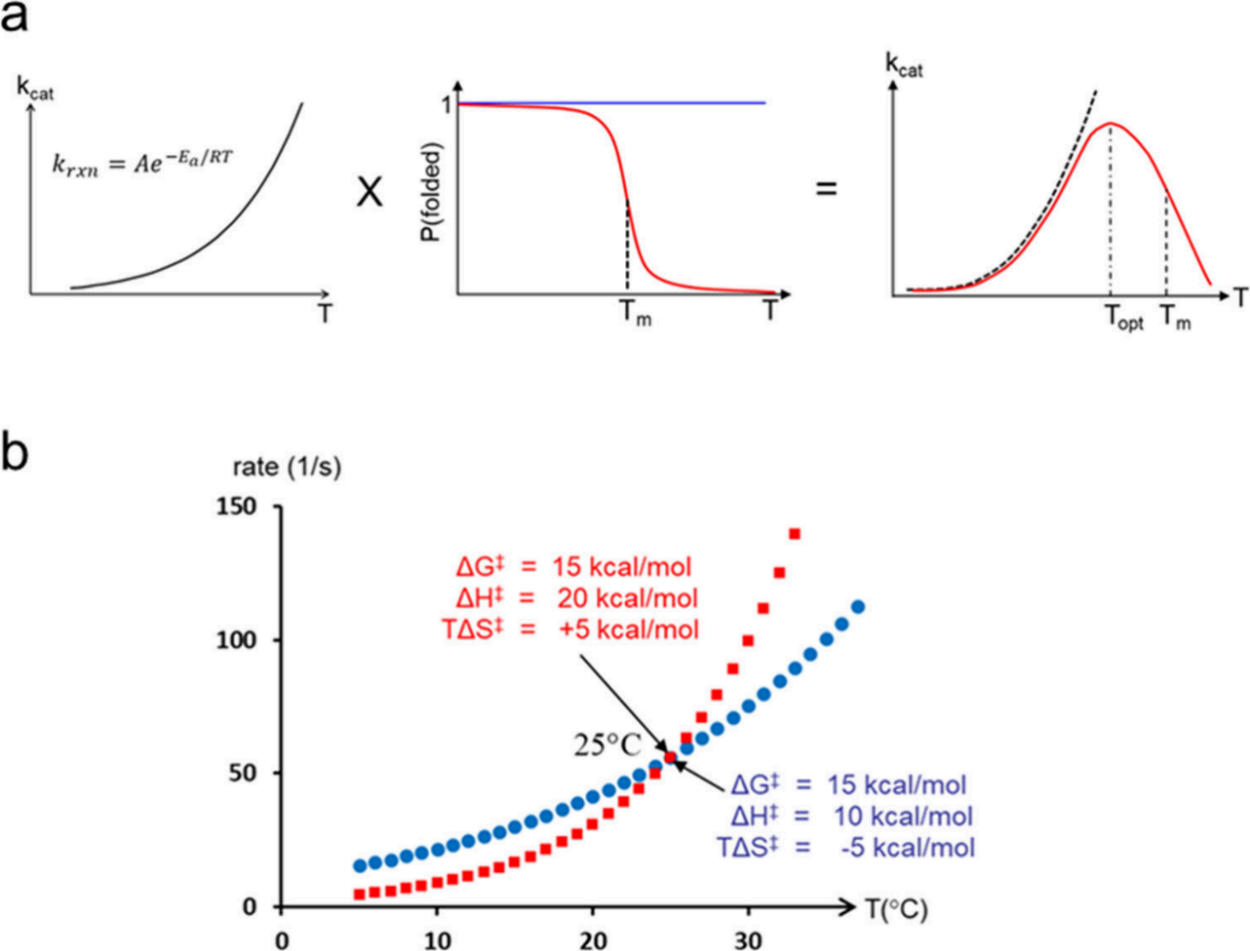
(**a**) Illustration of how an unfolding equilibrium naturally leads to a rate optimum with *T*_opt_ close to *T*_m_. (**b**) Illustration of how the temperature dependence of a hypothetical reaction with a free energy barrier of 15 kcal/mol at 25° is altered when enthalpy contribution is changed from 20 kcal/mol (red curve) to 10 kcal/mol (blue curve), with an opposing change in entropy contribution of 10 kcal/mol.

With regard to thermophilic enzymes, these can take advantage of the fact that reaction rates increase exponentially with increasing temperature (eq 1) and their main problem is to maintain stability at high temperature (Figure 4a). This can be seen from their 3D structures in terms of features that clearly counteract melting, such as shorter surface loops, more salt bridges, more hydrogen bonds, enhanced hydrophobic packing etc.^40^ The fact that protein denaturation eventually sets in as the temperature is increased naturally also leads to an optimum in the catalytic rate and usually  (Figure 4a). Mesophilic and thermophilic enzymes also work relatively closer to their melting point than psychrophilic ones. That is, the operational temperatures of typical cold-adapted enzymes are below 10 °C, while their *T*_*m*_ is well above room temperature. So, these enzymes do not have any problems with stability, but the question is how they can increase their catalytic rates at these low temperatures, where mesophilic enzymes generally have very low activity. Well, Somero’s finding from 1973^37^ provides the thermodynamic answer–they have reduced their activation enthalpy, partly at the expense of an increased entropy penalty (Figure 4b). As can seen from eq 1, it is the  factor that causes the rate decay at low *T*, so it makes perfect sense that the cold-active fish species have reduced this term in order to lift the tail of the rate curve.

How the shift of activation enthalpy–entropy balance has been achieved in cold-adapted enzymes has remained somewhat mysterious, in view of the fact that the enzyme structures appear very similar to orthologous mesophilic enzymes. In particular, the active sites are virtually identical when comparing psychrophilic and mesophilic enzymes.^40^ It was early suggested that the phenomenon could have something to do with protein flexibility and, particularly, with a higher degree of mobility in the active site region.^38,39^ A number of MD studies have, however, shows that active site residues usually have very similar mobility in psychrophilic and mesophilic enzymes, so that active site mobility does not seem to be the explanation.^40^

Since the reduction of  and a more negative  appears from biochemical kinetics to be the major adaptation in psychrophilic enzymes, it became clear to us that the only way to elicit the origin of the effect is to try to calculate these quantities from computer simulations.^40^ This was the main motivation for developing the scheme outlined above, where MD/EVB simulations are used to construct computational Arrhenius, from which  and  can be extracted with good accuracy.

## MD/EVB Simulations Explain the Enthalpy–Entropy Effect

**Figure 5:**
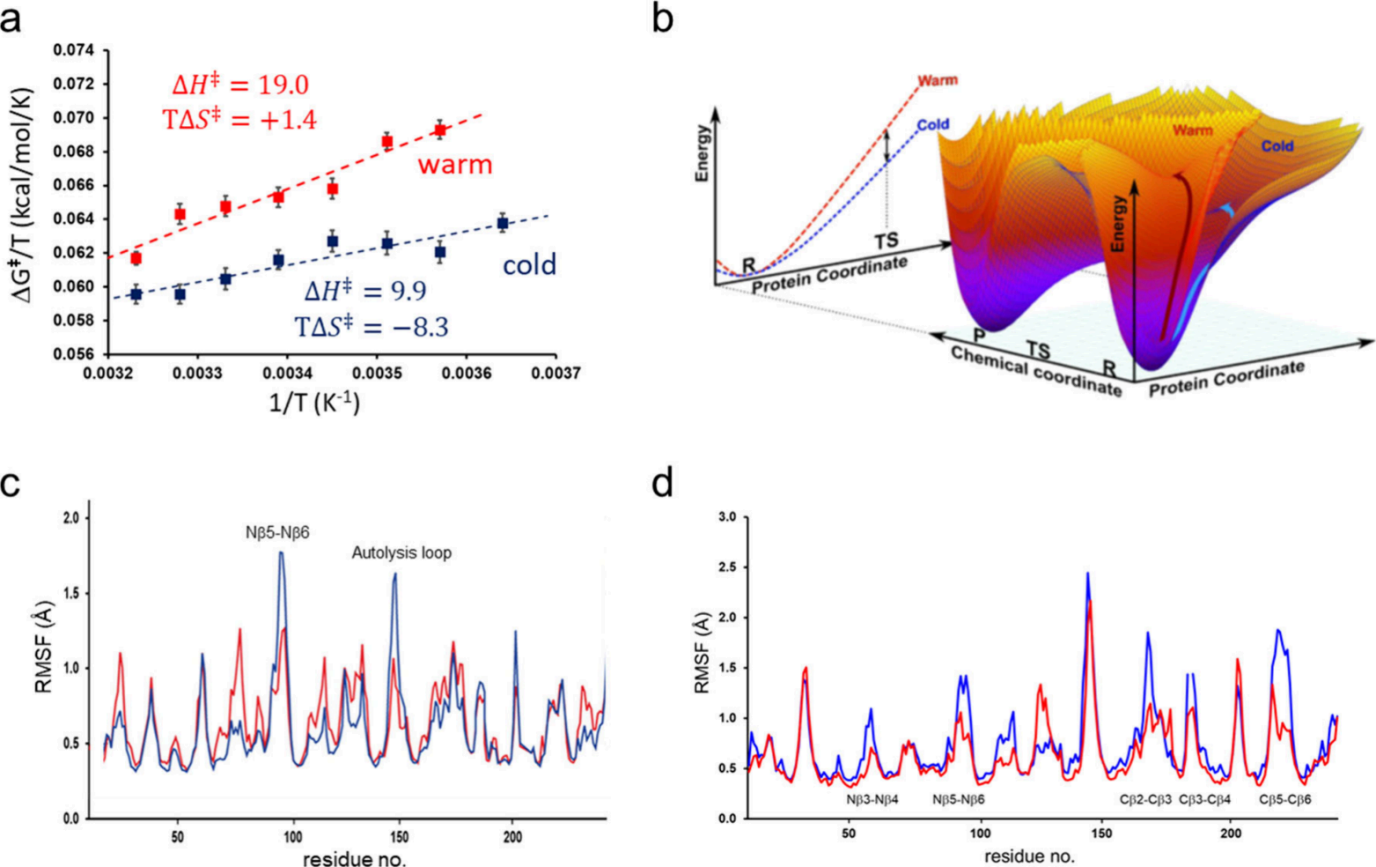
(**a**) Calculated Arrhenius plots for arctic salmon (blue) and bovine (red) trypsins.^42^ (**b**) Illustration of the concept of a softer protein potential in cold-adapted enzymes. The effective potential for displacing the protein coordinates from their reactant minimum to that of the TS is characterized by a smaller force constant than in the warm-active enzyme. (**c**) Calculated backbone RMSF averaged per residue for salmon (blue) and bovine (red) trypsins.^42^ (**d**) Calculated backbone RMSF averaged per residue for salmon (blue) and porcine (red) elastases.^43^ Mutated loops are indicated.

Remarkably, already the first attempts to calculate Arrhenius plots from MD/EVB simulations turned out to capture the above shift in the balance of  and . These studies involved cold- and warm-adapted citrate synthases and trypsins.^41,42^ That is, just by plugging in different starting structures in the simulations, the resulting Arrhenius plots came out with significantly different slopes, reflecting lower  values for the cold-adapted cases. (Figure 5a). In order to search for the structure origin of this effect, we first examined the energetics of the enthalpy term. If we denote the reacting fragments by *r* and the surrounding protein and solvent by *s*, the activation enthalpy is given by , apart from a negligible pressure–volume term. Here came the first surprise, when it turned out that it was primarily the  term that was most favorable for the cold-adapted enzymes. This therefore suggested that, in QM/MM language, it is not the QM or QM/MM interactions that are responsible for the observed effect, but rather interactions within the MM region. What it also then suggests is that the *response of the surrounding protein* to the perturbation caused by the chemical event is characterized by a more shallow effective potential for cold-adapted enzymes. That is, *U*_*ss*_ would be associated with a smaller force constant, implying that the surrounding protein is “softer” for these enzymes (Figure 5b).

The notion of a softer, or less stiff, protein naturally leads back to the ideas about protein flexibility playing a role cold-adaptation. Such ideas were, however, originally not particularly well formulated and were rather focused on heat lability of active site residues, as if the objective were to prevent “freezing” of the active site.^38,39^ What our results showed is that a generally softer protein directly translates into a smaller activation enthalpy. Moreover, this type of protein softness should be reflected in an increased flexibility if the effective force constant is lower, as the simulations implied. This was indeed found to be the case for all pairs of psychrophilic-mesophilic enzyme pairs studied to date.^40^ However, the increased flexibility of cold-adapted enzymes was found to be localized primarily to surface loops and not to active site residues (Figure 5c). The role of surface flexibility could be tested by making “computational mutations” where warm residues were inserted into the cold enzyme and vice versa. This was done for the two serine proteases trypsin and elastase, with the psychrophilic enzymes from arctic salmon and the mesophilic ones from pig.^42,44^ It turned out that all surface loop mutations than inserted a warm residue into the cold enzyme led to a higher  and lower backbone root-mean-square fluctuations (RMSF). Vice versa, all cold residues that were inserted into the warm enzymes led to the opposite effect, lower  and higher RMSF. As usual, the  term partly balances that of the activation enthalpy.^42,44^

The ultimate proof of the surface flexibility effect came from a series of wildtype salmon trypsin MD/EVB simulations where we just successively restrained the surface mobility from the outside and inward.^45^ This computational experiment, involving an 8-point Arrhenius plot for each set of restraints, led to the cold-adapted enzyme (salmon) acquiring mesophilic (pig) characteristics when residues beyond 18–20 Å from the active site were restrained. The values of  and  then changed from 9.9 and −8.3 kcal/mol, respectively, to 19.5 and +1.6 kcal/mol, with little change in  (the effect of the restraints was also seen with force constants as low as 1 kcal/mol/Å^2^).^42^ Another way of proving the surface flexibility effect is to just restrain particular surface loops that show higher mobility in the cold-adapted enzyme, and this was done both for trypsin and lactate dehydrogenase, again yielding the expected changes in  and .^45,46^ It should, however, be noted that the increased surface flexibility has nothing to do with the disputed concept of “dynamical effects” in enzyme catalysis, as it only pertains to modulation of the underlying potential energy surface.

## Cold-Adapted Enzymes May Become Inactivated before They Melt

It is not uncommon to observe that *T*_*opt*_ is significantly lower than *T*_*m*_ for cold-adapted enzymes, which implies that they are somehow inactivated before melting sets in.^24,38,39^ However, since their working temperature is much lower than both *T*_*opt*_ and *T*_*m*_, this is not really a problem for them. It should also be noted here that at their low operational temperatures there is presumably not much evolutionary pressure on stability, so that *T*_*opt*_ and *T*_*m*_ may have drifted toward lower temperatures just due to random mutations, which tend to cause this effect.^47^ Nevertheless, there has been considerable interest in explaining the origin of inactivation when . On a phenomenological level, any rate optimum can basically be described by temperature dependent, rather than constant, values of  and , which implies that there is a change in heat capacity, , between the reactant and transition state. That is, a rate curve with an optimum can usually be fitted to such a model with temperature dependent values of  and . However, such a model has little to say about the real microscopic origin of the optimum. For example, in the case that melting causes the optimum, , the origin of the effect is that the denatured “reactant state” starts to be significantly populated in the folding equilibrium as *T*_*m*_ is approached. Any such equilibrium where inactive conformations start to become populated will indeed cause an increase in *C*_*p*_ for the reactant state (and thus  but the concept of an activation heat capacity itself is not a very informative quantity, as far as the microscopic origin is concerned.^48^

## The Anomalous *T*-Optimum in a Cold-Adapted α-Amylase

**Figure 6:**
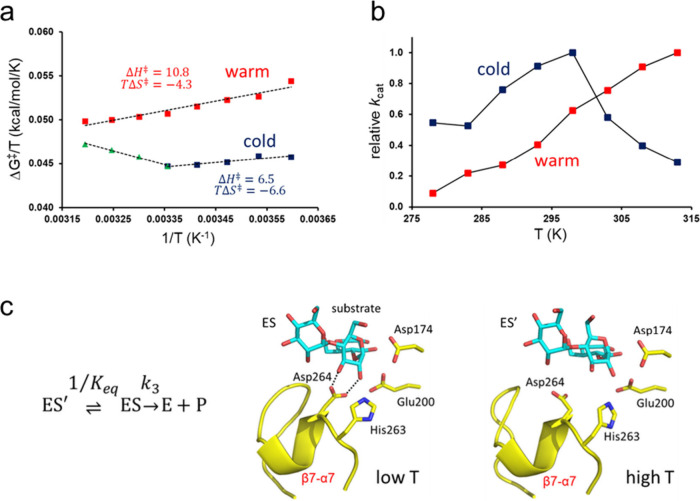
(**a**) Calculated Arrhenius plots for the reactions catalyzed by the α-amylases AHA (blue/green) and PPA (red). (**b**) Calculated *T*-dependence of the relative rate constants *k*_cat_ for AHA (blue) and PPA (red). (**c**) Average MD structures of active (ES) inactive (ES′) reactant states in AHA with H-bonds between Asp264 and the substrate indicated.

For cold-adapted enzymes with , the usual explanation of the inactivation has been that there is some partial or local unfolding going on, that is not easily detected by standard measurements of *T*_*m*_.^38,39^ A well characterized case is the psychrophilic α-amylase from the Antarctic bacterium *Pseudomonas haloplanktis* (AHA), which is a typical glycosidase acting on the α-1,4 bonds of starch molecules. In this case, the *T*-optimum at room temperature is about 15 °C lower than *T*_*m*_ as determined by fluorescence measurements.^24^ We thus constructed an EVB model for the rate-limiting glycosylation step based on DFT calculations on a reference reaction in water (Figure 2). This EVB parametrization was then used in simulations of both the cold-adapted AHA and the orthologous porcine pancreatic enzyme PPA. In the lower temperature region, the computed linear Arrhenius plots as usual show a lower  and a more negative  for the cold-adapted enzyme (AHA) than for the mesophilic PPA, with values compatible with those derived from experiment (Figure 6a).^23^

**Figure 7:**
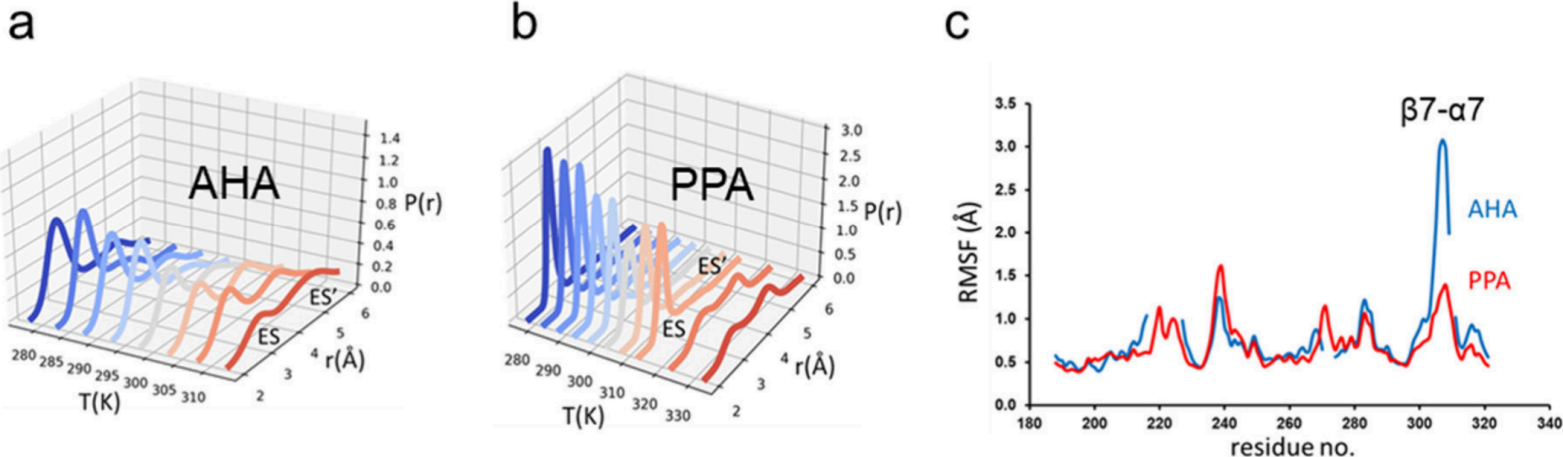
Calculated probability densities for the Asp264-substrate distance as a function of temperature for the (**a**) cold-adapted AHA and (**b**) warm-active PPA α-amylases. (**c**) Calculated average backbone RMSF per residue in the reactant state at 298 K for sequence region comprising the β7-α7 loop for AHA (blue) and PPA (red). PPA numbering is used due to sequence insertions.

Remarkably, however, we also see a clear break in the AHA plot which actually translates into a rate optimum around room temperature, just as seen experimentally (Figure 6b). This is the first example of that computer simulations can capture the *T*-optimum of an enzyme and it now allows us to examine the elusive origin of such effects. The explanation turs out to be rather simple, and somewhat similar to the idea of local unfolding, namely that a particular enzyme–substrate interaction involved in substrate binding breaks at around room temperature (Figure 6c). This leads to an increased population of the inactive state ES′ (or more precisely a state with higher  as the temperature is raised above the breakpoint. Once one knows what to look for, the effect can also be seen simply by calculating the probability densities of the active and inactive states as a function of temperature from plain MD simulations of the reactant state (Figure 7). The cold-adapted enzyme AHA enzyme evidently has a much less peaked distribution for the active ES state and ES′ starts to take over already at about 300 K. For the warm-active PPA enzyme, on the other hand, it is not until above 320 K that the population of ES′ starts to dominate. This is also in agreement with its reported temperature optimum at about 50 °C.^24^ The behavior of the critical β7-α7 loop region following the universally conserved His-Asp motif interacting with the substrate (Figure 6c) is also evident from its distinctly different mobility in AHA and PPA (Figure 7c).

**Figure 8:**
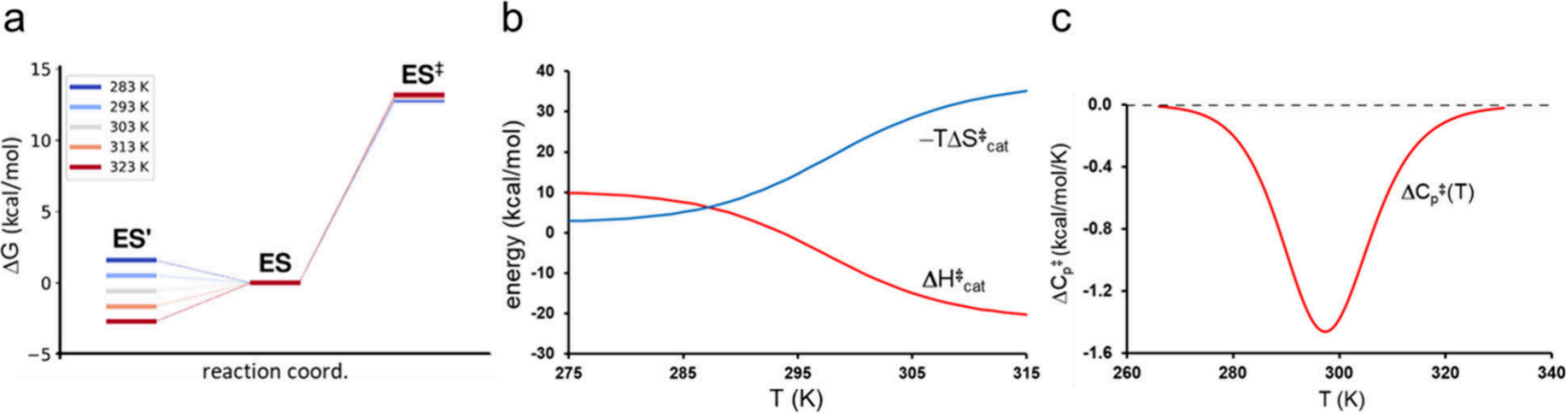
(**a**) Free energy diagram for the AHA reaction in the temperature range 283 K (blue) to 323 K (red). (**b**) Temperature dependence of the apparent activation enthalpy and entropy contributions obtained from the scheme of eq 3. (**c**) The corresponding activation heat capacity as a function of temperature.

We can further fit our calculated rates (*k*_*cat*_) for AHA as a function of *T* to an equilibrium model of the type
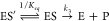
3where *K*_*eq*_ is the  equilibrium constant, *k*_*3*_ is the rate of the elementary chemical step from ES leading to products and *k*_*cat*_ is given by . This allows us to quantify the thermodynamics underlying *k*_*cat*_ as shown in Figure 8. Because the equilibrium has its midpoint at about 298 K, this is where ES′ starts to become lower in free energy (Figure 8a) and it is then the entropy penalty of moving from ES′ to ES that causes *k*_*cat*_ to decay. Here, the enthalpy associated with  equilibrium, , is large and positive since we are breaking an ionic hydrogen bond when moving to ES′. Conversely, when moving from ES′ via ES to P we get an apparent negative value of  at high *T* (Figure 8b). This may at first seem strange, but it only reflects the fact the activation enthalpy then is dominated by forming the ionic hydrogen bond in ES. The entropy penalty naturally goes in the opposite direction and eventually takes over. Furthermore, the temperature dependent activation heat capacity resulting from eq 3 can be directly calculated from  or  and its characteristic dip associated with a single reactant equilibrium is shown in Figure 8c (see refs (23) and (48) for further details). Interestingly, in the context of protein–ligand binding, it was also recently found that a somewhat analogous conformational equilibrium of the free ligands in solution can make a significant contribution to the observed negative binding heat capacity.^49^

## Can We Computationally Design the *T*-Dependence?

**Figure 9:**
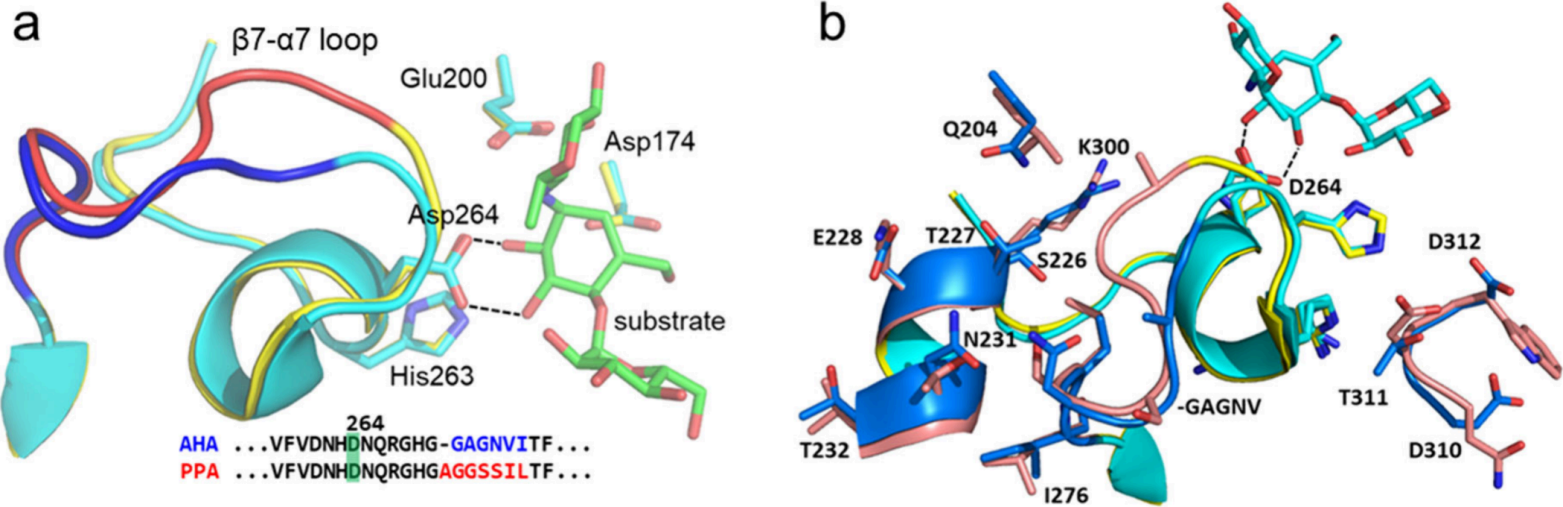
(**a**) View of the loop region following the universally conserved His-Asp motif in AHA (cyan/blue) and PPA (yellow/red). The catalytic residues Asp174 and Glu200 are also shown. (**b**) Mutated residues in the chimeric variants that interact with the β7-α7 loop.

A direct experimental proof of the cause of the anomalous *T-*optimum in the cold-adapted amylase would be to redesign the enzyme temperature dependence based on the computational results. The idea is then to shift the  equilibrium toward ES and to dampen the fluctuations of the β7-α7 loop. To this end, we computationally constructed a series of chimeric enzymes by inserting residues from PPA into AHA.^50^ The simplest variant (Chimera 0) just replaced the –GAGNV loop motif in AHA by AGGSSI that is found in PPA (Figure 9a). Four additional chimeras (Chimera 1–4) containing an increasing number of mutations surrounding the loop were also made, where Chimera 4 had a total of 16 mutations from PPA, including the loop sequence above (Figure 9b). The shift of the  equilibrium at 25 °C was then monitored by plain MD simulations and, while already Chimera 0 showed some stabilization of ES, the effect was most pronounced for Chimera 4. Subsequent MD/EVB simulations also of the catalytic reaction for that variant also predicted a shift of the temperature optimum of more than 10 °C.^50^

The genes for AHA, PPA and Chimeras 0 and 4 were then bought and expressed and both steady-state kinetics and temperature ramping experiments were carried out with the relatively slow substrate 2-chloro-4-nitrophenyl-α-D-maltotrioside (CNP-G3).^50^ These results indeed showed that Chimera 0 moved the optimum upward by 2 °C while Chimera 4 produced a larger effect of 6 °C, as expected from the simulations. Both Chimeras 0 and 4 are less active than wildtype AHA at 25 °C (68% and 17%, respectively). However, Chimera 4 was found to be faster than both AHA and PPA at moderately higher temperatures. The crystal structure of Chimera 4 was also determined and showed that the β7-α7 loop had indeed moved from its position in AHA toward that in PPA.^50^ Hence, the computational prediction that the β7-α7 loop and the critical Asp264-substrate interaction control the temperature optimum in the cold-adapted α-amylase was confirmed by the experiments.

## Conclusions

We have outlined here how the MD/EVB methodology can be effectively used to obtain thermodynamic activation parameters for enzyme reaction from simulations of the catalytic reaction. The key idea is then to evaluate the temperature dependence of the rate-limiting activation free energy barrier and, from these results, compute Arrhenius plots in analogy with the experimental approach. Because the extraction of  and  from linear regression requires very high precision in the calculated values of , it is necessary to carry out extensive sampling by MD simulations. For this reason, there is at present not really any other method that can compete with the EVB model, although this may well change in the future.

Besides the obvious application of the method to try to answer long-standing questions regarding enthalpic versus entropic catalytic effects in specific enzymes, there are also very interesting evolutionary questions regarding enzyme adaptation that can be addressed. In particular, temperature adaptation of enzyme catalysis is a hot topic, as we now have access to large amounts of both structure and (particularly) sequence information for enzymes from differently adapted species. It should perhaps also be pointed out that it is difficult to predict solely from sequence whether a given enzyme is cold-adapted or not.^39^ Moreover, not all enzymes from psychrophilic species show cold-adapted characteristics since they may not be limiting for survival and growth and biochemical experiments are thus essential in this regard. As discussed above, enzyme adaptation to different environmental temperatures is characterized by distinct relationships between activation enthalpy and entropy, which makes these quantities very relevant to calculate.^37^

We have reviewed here how a number of MD/EVB studies of cold-adapted enzymes directly capture the activation enthalpy–entropy shift compared to orthologous mesophilic enzymes. Moreover, these simulations clearly show that the flexibility of protein surface loops is a major determinant of the effect, as there is a direct connection between the stiffness/softness of the effective protein potential energy function and the enthalpy and entropy contributions to the activation free energy of the catalyzed reaction. It may thus be tempting to try to analyze enzyme temperature adaptation by just monitoring protein flexibility from plain MD simulations. However, such an approach misses the point that, in the end, it is the catalytic rate at a given temperature that tells whether the enzyme is adapted to these conditions, or not. Hence, if you do not calculate the activation free energy of the chemical reaction you are not really in business.

The ability to use computer simulations to study and predict the temperature dependence of enzyme reactions also opens up the possibility of using calculations to redesign this property by mutagenesis. In particular, it is of considerable biotechnological interest to improve the catalytic activity outside of the normal temperature range of certain enzymes and to be able to shift their catalytic optima. In the case of the cold-adapted α-amylase we have shown that this indeed possible and we could also identify the structural origin of its anomalously low optimum. That work also illustrates a problem that might be connected with taking a cold-adapted enzyme and trying to move its optimum upward, namely that it may be accompanied by a loss of activity at low temperature. How to overcome such challenges by computational design is certainly an interesting issue that deserves further study.
